# Late-Presenting Septic Arthritis of the Hip in Children: Variations in Presentation and a Review of 25 Hips After Surgical Debridement

**DOI:** 10.7759/cureus.47717

**Published:** 2023-10-26

**Authors:** Suresh Chand, Shubham Srivastava, Syed Faisal Afaque, Ajeet Yadav, Vikas Verma, Shakeel Qidwai, Ajai Singh

**Affiliations:** 1 Paediatric Orthopaedics, King George's Medical University, Lucknow, IND; 2 Orthopaedic Surgery, King George's Medical University, Lucknow, IND; 3 Orthopedics, Mahamaya Rajkiya Allopathic Medical College, Akbarpur, IND

**Keywords:** joint infection, septic sequalae, septic capital slip, hip dislocation, septic arthritis

## Abstract

Introduction: The septic arthritis of the hip (SAH) is one of the most common musculoskeletal infections occurring in pediatric populations requiring urgent intervention. This study discusses the myriad of clinical and radiological presentations of late-presenting SAH in children and the outcomes of surgical management.

Methods: After ethical approval, we did retrospective reviews of children treated for late-presenting SAH (after five days of symptoms). We excluded late cases with established sequelae. We recorded age, duration of symptoms, clinical evaluation, and radiographs. We evaluated the final results clinically and radiologically.

Results: Twenty-four patients with 25 hips were eligible for evaluation. At presentation, all had decreased or painful hip movements, but none had a fever. Radiographs revealed the following changes: hip dislocation (four), capital femoral slip (seven), proximal femur/neck osteomyelitis (six), pathological fractured neck femur (two), iliac osteomyelitis (two), and early arthritic changes (two). Hip arthrotomy was done in all cases. Frank pus was found in 21 (84%) cases. Cases with capital slip and fractured neck femur required fixation with two smooth K-wires. Methicillin-resistant Staphylococcus aureus (MRSA) was isolated in three patients and tuberculosis in two cases. Clinical outcomes showed 14 patients with poor outcomes, eight with fair, and two with good. Avascular necrosis (AVN) of the femoral head was noted in 14 hips and complete femoral head resorption in nine.

Conclusions: The late-presenting SAH in children has a myriad of presentations including dislocation and capital slip with unsatisfactory outcome. However, ongoing local infective processes may necessitate debridement. With limited salvage options available at the sequelae stage, awareness and training for early diagnosis and treatment may be the best way to improve the scenario. We recommend future multicenter randomized studies of predictive factors and indications of arthrotomy in late presenters.

## Introduction

Childhood musculoskeletal infections have a variety of presentations and are significant causes of morbidity and mortality [1]. The incidence of septic arthritis is approximately one to five per 100,000 children [2]. More than one-third of cases of septic arthritis in children involve the hip joint, and almost half of those children are under two years old [3].

Several diagnostic criteria have been proposed and used for diagnosis of septic arthritis [4]. However, their reliability and generalizability are debatable [5,6]. Septic arthritis of the hip (SAH) is an important diagnostic and management challenge in children, especially in resource-limited countries. It is a well-accepted fact that SAH in children is an orthopedic emergency, and cartilage destruction can be observed as early as eight hours after joint infection [7,8]. To prevent devastating damage to the joint, early arthrotomy and appropriate antibiotic therapy are desirable. In the literature, there is no clearly defined demarcation for the duration of delay. However, most authors agree that the delay in treatment beyond five days of symptoms is associated with unsatisfactory outcomes [9-11]. The residual damage in a child’s hip joint is more pronounced than in other joints [12]. This further emphasizes the role of early diagnosis and management.

Because of various factors, the timely diagnosis and management of SAH is often missed or delayed, particularly in low- and middle-income countries [2]. At a tertiary care institute, it is not uncommon to receive patients in subacute phases or late stages of the disease with prior inadequate or no treatment. Many articles discuss the presentation and management of acute SAH in children or the description of sequelae and its salvage [13-16]. However, there is scant literature dedicated to the delayed presentation scenario and its management [11]. This study discusses the myriad of clinical and radiological presentations of late-presenting SAH and the outcomes of surgical management in children.

## Materials and methods

This retrospective study was approved by the Institutional Ethical Committee, and we obtained written informed consent from the children’s parents. We retrospectively evaluated cases of children with SAH who presented to our hospital between January 2019 and December 2021. We defined “late-presenting septic arthritis of the hip” as patients who underwent surgical intervention after >five days of duration of symptoms [9-11]. Cases with late-presenting SAH, with or without the involvement of other parts or joints, were included in the study. We excluded late cases of patients with no active infection (clinico-radiologically) and with established sequelae of arthritis and cases of patients undergoing salvage procedures. Clinico-radiological features that we considered as active infection in late-presenting cases included the following:

1) Hip pain with refusal to use limb actively, and

2) Local ultrasound (USG) or magnetic resonance imaging (MRI) suggestive of collection in the hip and/or proximal femur region.

Management Protocol

After the arrival of a patient in a hospital, we obtained, in addition to routine blood investigations, quantitative C-reactive protein (CRP) and erythrocyte sedimentation rate (ESR). We did not use preoperative blood culture because, as part of a tertiary care center, most of the referred cases were already under antibiotic cover at a previous treatment facility for a variable duration. We obtained a radiograph of the pelvis with both hips anteroposterior with a frog leg lateral view. We ordered additional X-rays in cases where we suspected other joint involvement. We performed high-resolution ultrasound (HR-USG) for every patient to document the amount and extent of collection, if any. We ordered an MRI where extra-articular pelvic involvement was suspected.

Broad-spectrum intravenous antibiotics were begun immediately after admission. After consent, patients were taken to the operating room under general anesthesia. In the supine position, all patients underwent hip arthrotomy via the Smith-Peterson approach using a bikini incision. A hip joint capsulotomy was done. Thorough joint debridement and lavage were done. A negative suction drain was applied. In cases where osteomyelitis changes were seen, proximal femoral decompression was done using a separate lateral incision. Adductor tenotomy was performed in cases where hip abduction was limited.

Operative findings were noted. Samples for culture and sensitivity and a histopathological biopsy were obtained from each patient. In cases with associated hip dislocation/subluxation, reduction of the hip joint was done. Cases with capital femoral slips or pathological fractured neck femur were reduced gently and fixed with two smooth K-wires. In all cases, postoperative hip spica was given for 8-10 weeks. A local wound check was done after 48 hours using a small window over the spica. Because of the doubtful reliability of blood parameters in late-presenting cases, we looked for surrogate markers for healing infections: healing of surgical wounds, improvement in pain, and active hip use. Any K-wires were removed after 8-10 weeks. After the removal of spica, patients were given a hip abduction brace for the next six weeks with gradual weaning and range of motion therapy. This was followed by gradual weight bearing as tolerated.

We collected age at the time of presentation, duration of symptoms before undergoing arthrotomy, clinical examination findings, and radiographs. The final clinical results were graded by Moon’s criteria [17]. An “excellent” outcome was defined as a pain-free hip and normal ambulation, sitting possible in any position; “good” as slight pain, occasional, no compromise in activities, uneasy squatting; “fair” as mild pain, no effect on average activities, rarely moderate pain with unusual activity, some limitation in cross legged and squatting; and “poor” as moderate and marked pain, limitation of ordinary activity, and serious limitation of activities.

The final radiological evaluation was done using the classification provided by Choi et al. and Johari et al. [14,15]. Avascular necrosis (AVN) at the final follow-up was graded using the Kalamchi classification [18]. The results were analyzed using descriptive statistics. Discrete data were summarized as mean (SD), and categorical data were summarized as proportions and percentages. Statistical analyses were performed using Statistical Product and Service Solutions (SPSS) (version 23.0; IBM SPSS Statistics for Windows, Armonk, NY).

## Results

In our institute, thirty-two children with late-presenting SAH were surgically treated from January 2019 to December 2021. Eight patients were lost to follow-up after arthrotomy. One patient had bilateral-hip-joint involvement. Therefore, 24 patients with 25 hips were eligible for evaluation of the final results. Basic details of included cases are summarized in Table 1.

**Table 1:** Basic details of cases included.

Total number of cases	N = 25 hips (girls, 14; boys, 10)
Mean age	55.5 months (range 2-114 months)
Mean delay in presentation	43.25 (range 5-87 days)
Mean follow-up period	21.79 months (range 12-38 months)

At presentation, all had complaints of decreased or painful hip movements and inability to walk (in a previously walking child). None had a fever. Findings as per modified Kocher’s criteria are summarized in Table 2.

**Table 2:** Modified Kocher’s criteria at presentation among the cases included.

	Mean	Standard deviation	Median	Minimum	Maximum	Valid "n"
Temperature	37.68 (°C)	0.36	37.70	37.00	38.20	24
Total leucocyte count	12,960.58 (cells/mm^3^)	4,391.16	12,650.00	5,204.00	21,300.00	24
C-reactive protein	18.08 (mg/L)	17.33	12.00	5.00	77.00	24
Erythrocyte sedimentation rate	29.67 (mm/hr)	11.66	32.00	12.00	48.00	24
Pain/limp	Present in all cases					

In our series of cases with late-presenting SAH, six children (25%) had one predictor, 10 children (41.6%) had two predictors, eight children (33.3%) had three predictors, and none had four or five predictors under the modified Kocher’s criteria. On admission, the raised values (as per modified Kocher’s) of total leucocyte count (TLC) were noted in 62.5% of the cases, while raised CRP and ESR were noted in 25%.

**Figure 1:**
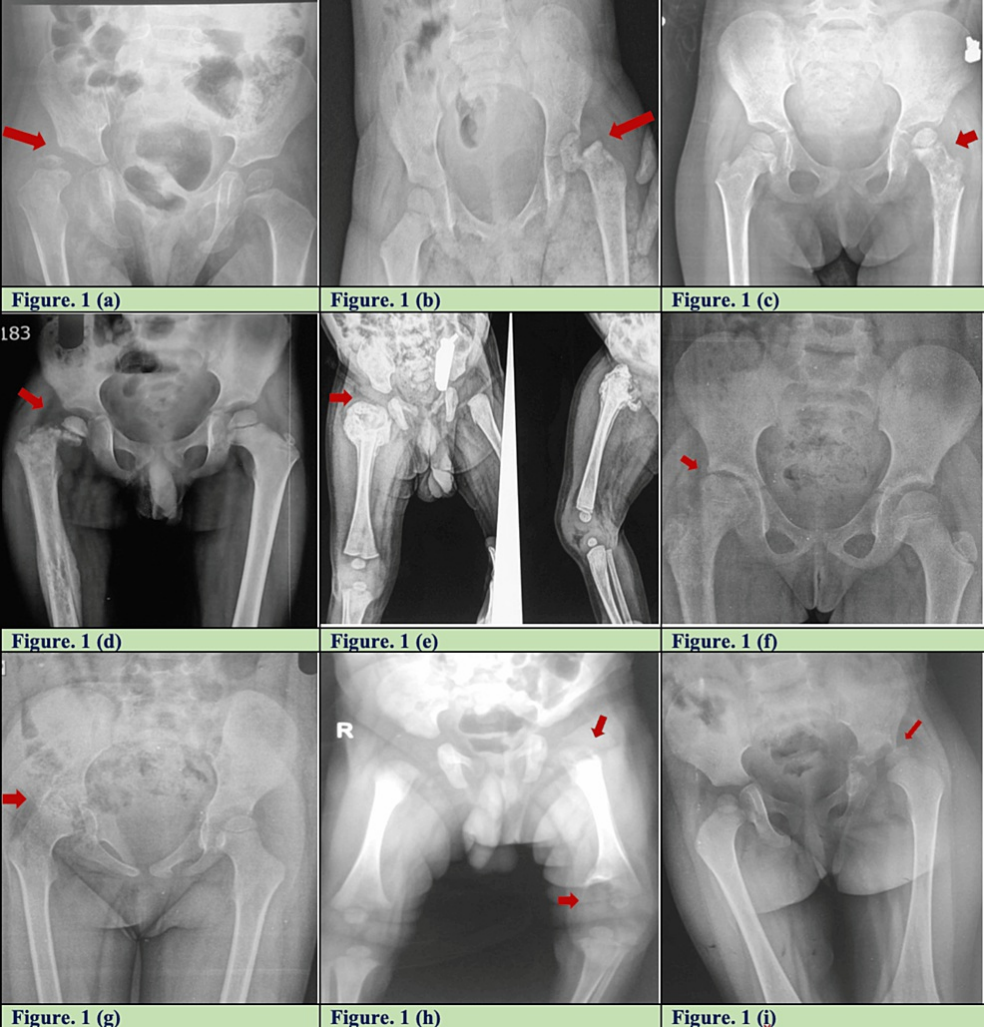
Variations in the radiological appearance of late-presenting septic arthritis of the hip in children. 1(a) Right SAH with septic dislocation, 1(b) left SAH showing capital slip with dislocation, 1(c) left SAH with osteomyelitis of proximal femur and shaft, 1(d) right SAH with pathological fracture neck femur with osteomyelitis shaft femur, 1(e) right SAH with osteomyelitis proximal femur showing hyperplastic callus, 1(f) right SAH with early arthritis/AVN, 1(g) right SAH with osteomyelitis of acetabulum, 1(h) left SAH with osteomyelitis of proximal and distal femur, and 1(i) left SAH with subluxation and osteomyelitis acetabulum. SAH: Septic arthritis of hip; AVN: Avascular necrosis of femoral head

Plain radiographs (X-ray of the pelvis with both hips) done at the time of presentation revealed a myriad of presentations in all but two cases. The following changes were noted on X-ray, alone, or in various combinations: hip dislocation (four cases), capital femoral slip (seven), proximal femur/neck osteomyelitis (six), pathological fractured neck femur (two), iliac/acetabular osteomyelitis (two), and early arthritic changes (two) (Figure 1).

In all 25 hips, high-resolution ultrasonography (HR-USG) reports were available, out of which 18 reports indicated moderate hip-joint effusions, and, in seven cases, hip-joint synovitis with minimal effusion was reported. MRI confirmed the hip joint effusion in all these cases. MRI revealed additional femoral (nine hips), iliac/acetabular osteomyelitis (three hips), and psoas abscess (two hips). Before presenting to us, all had received a range of antibiotics (intravenous or oral) of variable duration from local pediatricians or general practitioners. 

In all 25 cases, hip arthrotomy was done. Intraoperatively, frank pus was found inside the hip joint in 21 (84%) cases, and granulation tissue was noted in the remaining. Using a separate lateral approach, nine patients underwent femoral decompression for proximal femoral osteomyelitis. In five patients, other joints were involved in addition to SAH. In three patients, the ipsilateral knee joint was involved. In one patient, each shoulder and elbow joint were involved. All involved joints underwent arthrotomy along with the hip joint. In two patients, their psoas abscesses were drained with a separate incision over the iliac crest. Seven cases with capital slip and two cases with pathological neck fracture required fixation with two smooth K-wires. Six patients required percutaneous adductor tenotomy to improve abduction. Postoperatively, all patients were given hip spica.

Methicillin-resistant Staphylococcus aureus (MRSA) was isolated in three (12%) patients. In two patients, acid-fast bacilli were detected and were rifampicin-sensitive mycobacterium tuberculosis (TB). In the remaining cases, the culture reports were sterile. The histopathological report was inconclusive in most cases, except the cases with M. tuberculosis. All patients were given five to seven days of intravenous antibiotics (vancomycin). Twenty-three patients (92%) were followed by six weeks of oral antibiotic (linezolid/clindamycin). Two patients with positive TB reports were started on anti-tubercular therapy (ATT) based on INDEX-TB guidelines [19]. Postoperatively, wound dehiscence was noted in three cases, two of which were tubercular and healed spontaneously after ATT was started. One patient with sterile culture had wound dehiscence with persistent discharge and was given extended intravenous vancomycin and underwent re-debridement. Pin-site infection was noted in four and plaster sores in two cases, which healed with dressings. By seven to eight weeks after arthrotomy, all patients (except those with TB) had normal blood parameters (TLC, CRP, ESR), and their surgical wounds were healed, hip pain was markedly reduced, and infections were considered controlled. Tubercular cases underwent 12 months of ATT before being declared cured.

At the final follow-up, all patients were using the affected limb actively. Clinical shortening was present in 18 patients, with an average of 1.67 cm. The Trendelenburg and telescopy test was positive in 15 and 12 patients, respectively. Clinical outcomes assessed by Moon’s criteria were poor in most patients (n = 14, 56%), with eight having fair and two having good outcomes. None of the patients had an excellent outcome. Radiological assessment of outcomes at the final follow-up showed an AVN of the femoral head in 14 hips. The final outcome of septic hip assessed by both the Choi and Johari classification is shown in Table 3.

**Table 3:** The radiological outcome at the final follow-up. AVN = Avascular necrosis of the hip.

Choi staging [14]		Johari staging [15]		AVN status (Kalamchi) [18]	
Stage	No. of hips	Stage	No. of hips	Stage	No. of hips
1	4	1	7	1	4
2A	5	2	3	2	2
2B	3	3A	0	3	1
3A	2	3B	2	4	7
3B	2	4	11		
4A	7	5	2		
4B	2				

**Figure 2:**
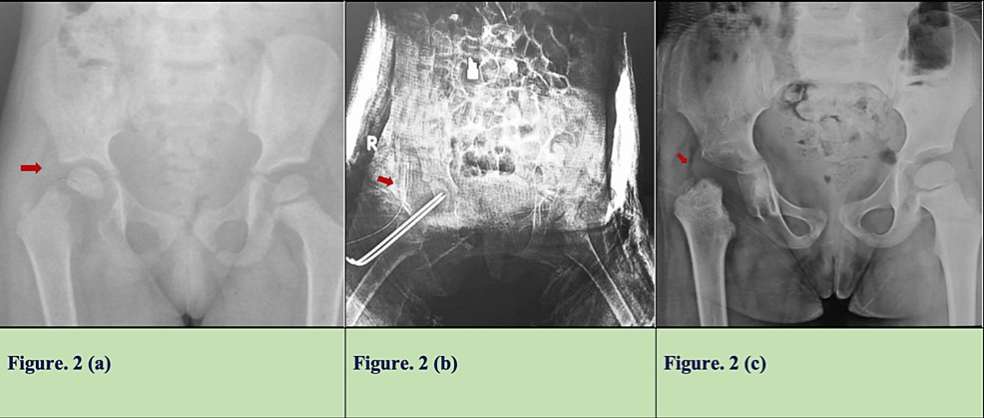
A three-year-old child with septic hip arthritis with capital slip, (b) postoperative image after arthrotomy and k-wire fixation of slip, and (c) follow-up showing complete resorption of capital epiphysis (Choi type 4A).

There was complete femoral head resorption in nine cases (this included seven cases with capital slip and two cases with fractured neck femur) (Figure 2).

The average duration of noticing femoral head resorption on X-ray was five months (range of two to seven months). Six hips were having concomitant acetabular arthritic changes at the final follow-up.

## Discussion

As SAH is a rapidly progressive disease, it is difficult to categorize cases as acute or late based on the duration of symptoms. However, few studies in the literature have found SAH lasting beyond five days to be associated with poor clinical and radiological outcomes. Our retrospective study discusses the clinical and radiological findings in children with late-presenting SAH and the outcomes of surgery. To our knowledge, ours is the first study intended to explore the myriad of presentations in late-presenting SAH in children.

In our study, at the time of presentation, none of the patients had a fever, and only one-fourth had positive ESR/CRP predictors as per the modified Kocher’s criteria. This highlights the poor reliability of Kocher’s criteria in late-presenting SAH [4]. A high index of suspicion along with clinical examination is key to detecting these cases. A USG was able to detect effusion in all these late-presenting cases. However, none of the patients had severe effusion. An MRI was helpful in detecting associated unrecognized psoas abscess in two cases and proximal femoral osteomyelitis in three cases. If resources permit, we suggest an MRI should be performed on all children suspected of a late-presenting SAH.

Many authors have described the classification for variations of sequelae of SAH [13-16]. However, there is a paucity of literature on variations in late-presenting cases with active infection. On admission, we noted a myriad of presentations on X-ray, as shown in Figure 1. Based on X-ray findings, Table 4 shows variations of late-presenting SAH in children.

**Table 4:** Variations in late presenting septic arthritis of the hip joint in children (based on X-ray findings at the time of admission).

Group		Description
1	No X-ray changes	Only clinical signs (pain/ limp/ limitation of motion)
2A	Early X-ray changes (with contained hip)	Proximal femoral osteomyelitis with or without early arthritis
2B		Proximal femoral osteomyelitis with pathological fracture neck femur
3A	Hip dislocation	Hip dislocation without proximal femoral osteomyelitis
3B		Hip dislocation with proximal femoral osteomyelitis
4A	Capital slip	Capital femoral slip without proximal femoral osteomyelitis
4B		Capital femoral slip with proximal femoral osteomyelitis
4C		Capital femoral slip with joint dislocation with or without femoral osteomyelitis

We acknowledge that this is an attempt to tabulate many variations, and perhaps many more combinations are possible. In this study, all but two patients had positive X-ray findings at presentation. With the history of fever (although none had fever at presentation to us) and painful hip with effusion on USG, we considered that all had an active infection requiring debridement. The presence of pus intraoperatively in most of the cases reconfirmed the active state of infection and that they were not at the sequelae stage yet.

Some authors have described hip dislocation with the presence of CFE at the sequelae stage [15,16]. Agarwal et al. described acute septic dislocation in 14 children treated by reduction at the time of arthrotomy [20]. Ngom et al. described some good results with recent (<3 weeks) septic dislocations [21]. In our study, we noticed hip joint dislocation in four cases, with a duration of symptoms as early as two weeks. All these patients underwent arthrotomy and debridement followed by hip spica application. Capsulorrhaphy was not done after arthrotomy in view of active infection. As of the latest follow-up, all these hips were stable and contained. At a short follow-up, our findings in this subset of patients agreed with Agarwal et al. and Ngom et al. [20,21]. With the small sample size, it is difficult to correlate dislocation in SAH with duration of symptoms or any other factor. Nonetheless, a high index of suspicion is necessary to diagnose and treat or prevent dislocation in cases with SAH. At our institute, all patients with acute SAH <five years undergo hip spica application after arthrotomy to prevent postoperative subluxation or dislocation. A Pavlik harness is considered in neonates or small babies in NICUs with poor systemic conditions. Patients >five years of age are given skin traction at least for six weeks with a hip in abduction. However, patients with late-presenting SAH (all cases in our study) underwent spica application.

There have been reports of proximal femoral epiphyseal separations or septic slips after SAH in neonates and infants [22-25]. However, they are rare, especially in children older than two [23,26]. A few reports have described a delayed capital slip after SAH [27]. Although the treatment of septic slip with SAH is associated with unpredictable and poor prognosis, it is worthwhile attempting to salvage the slip after arthrotomy. In our study, we noticed a capital slip with SAH in seven cases. One patient had associated hip dislocation along with a septic slip. It appears that with persistent infection, hip dislocation may have progressed to septic capital slip. All patients underwent k-wire fixation after debridement. At the last follow-up, all these patients had complete resorption of the femoral epiphysis with poor outcomes as per Moon’s criteria and type 4A as per Choi et al. Two patients with associated fractured neck femur also had resorption of the femoral head with a poor outcome.

Overall, S. aureus is the most common pathogen that causes septic arthritis. However, culture-negative septic arthritis has been reported in up to two-thirds of the cases [28]. K. kingae has been reported to be the most commonly isolated organism from PCR-positive confirmed septic arthritis [29]. Routine cultures performed at most facilities could detect only one-third of these cases. In our retrospective study, the culture and sensitivity reports were negative in 84% of the cases. The late presentation with a history of antibiotic treatment before admission may be a contributing factor in our heterogeneous group of cases.

Delayed presentation of SAH has been associated with unsatisfactory outcomes [11]. In our study, more than half the cases had poor outcomes at final follow-up as per Moon’s criteria, and 36% of cases had complete head resorption as late as seven months postoperatively. Thus, we recommend regular and frequent follow-up in the treatment of SAH.

In our study, we noticed a delay of up to three months (mean delay of 43 days). We acknowledge that this is based on the recall of parents and may not depict the disease duration precisely. We did not include details of treatment before admission. However, all patients were provided with antibiotics of variable duration for initial symptoms of fever, pain, or refusal to use limbs. This highlights the need for a high index of suspicion and awareness among general and pediatric practitioners to detect SAH. The other reasons for delay, apart from a delay in diagnosis, may include unawareness of parents regarding the emergency nature of septic arthritis, nonavailability of transport to the hospital from remote areas, and nonavailability of centers performing arthrotomy in children, and poverty.

Describing the exact cutoff for the duration to define the infection activity in SAH will always be difficult. Moreover, considering the unsatisfactory outcome in most cases of late-presenting SAH, one may argue in favor of expectant management. We performed an arthrotomy up to a delay of three months, and the presence of effusion on USG and pus intraoperatively justified the procedure. Moreover, two cases were diagnosed as tubercular SAH, which would have been missed with expectant management. In the future, randomized prospective studies may be helpful in devising predictive factors for outcomes and indications for arthrotomy in late-presenting SAH.

Our study has a few limitations. The small sample size collected retrospectively might not represent disease in the general population. The cases were heterogeneous, and there was no standard initial treatment before admission. Moreover, the duration of symptoms was based on parents’ understanding and recall, and the definition of delayed (>five days) is a bit arbitrary. The response to arthrotomy and antibiotics in our study was largely subjective in the absence of reliable parameters for late-presenting SAH. Because of resource constraints, we did not repeat MRI or USG at the end of the treatment or final follow-up. We could not perform a regression analysis for various factors affecting the outcomes because of the small sample size of the heterogeneous nature of this retrospective study. We recommend future prospective studies with more cases followed till maturity.

However, our study has exclusively described the various manifestations of late-presenting SAH. We have noticed that it is not uncommon to have septic hip dislocation or septic capital slip in SAH, with septic slip having the poorest outcome. The progressive nature of damage and delayed manifestations highlight the need for regular follow-up. Moreover, an unsatisfactory final outcome strengthens the call for aggressive and urgent treatment in SAH. Despite guarded prognosis in late-presenting cases, it may be worthwhile to perform debridement because our study revealed the presence of pus or granulation tissue in most of these cases. Considering the limited options available for sequelae of SAH, our study reemphasizes that the best bet seems to be at the prevention level. To prevent a delay in diagnosis, there is a need to increase awareness among general practitioners and pediatricians regarding SAH, as expressed in the well-known saying “Prevention is better than cure.” For SAH, it may be paraphrased as “Early diagnosis and treatment is better than dealing with sequelae.”

## Conclusions

The late-presenting SAH in children has a myriad of presentations including septic dislocation and septic capital slip. A high index of suspicion with clinical and radiological examination is crucial because of the poor reliability of Kocher’s criteria in delayed presentation. The clinical and radiological outcomes of this subset are unsatisfactory. However, an ongoing local infective process may necessitate debridement. With limited salvage options available at the sequelae stage, awareness and training for early diagnosis and treatment may be the best way to improve the scenario. Moreover, we recommend large, multicenter, and randomized studies to study predictive factors and indications of arthrotomy in late presenters.
